# Injuries from Non-Retention in Gillnet Fisheries Suppress Reproductive Maturation in Escaped Fish

**DOI:** 10.1371/journal.pone.0069615

**Published:** 2013-07-24

**Authors:** Matthew R. Baker, Penny Swanson, Graham Young

**Affiliations:** 1 School of Aquatic and Fishery Sciences, University of Washington, Seattle, Washington, United States of America; 2 Northwest Fisheries Science Center, NOAA Fisheries Service, Seattle, Washington, United States of America; 3 Center for Reproductive Biology, Washington State University, Pullman, Washington, United States of America; Monash University, Australia

## Abstract

Exploitation of fisheries resources has unintended consequences, not only in the bycatch and discard of non-target organisms, but also in damage to targeted fish that are injured by gear but not landed (non-retention). Delayed mortality due to non-retention represents lost reproductive potential in exploited stocks, while not contributing to harvest. Our study examined the physiological mechanisms by which delayed mortality occurs and the extent to which injuries related to disentanglement from commercial gear compromise reproductive success in spawning stocks of Pacific salmon (*Oncorhynchus spp.).* We found evidence for elevated stress in fish injured via non-retention in gillnet fisheries. Plasma cortisol levels correlated with the severity of disentanglement injury and were elevated in fish that developed infections related to disentanglement injuries. We also analyzed sex steroid concentrations in females (estradiol-17β and 17,20β-dihydroxy-4-pregnen-3-one) to determine whether non-retention impairs reproductive potential in escaped individuals. We demonstrate evidence for delayed or inhibited maturation in fish with disentanglement injuries. These findings have important implications for effective conservation and management of exploited fish stocks and suggest means to improve spawning success in such stocks if retention in commercial fisheries is improved and incidental mortality reduced.

## Introduction

### Salmon fisheries and non-retention

**Figure 1:**
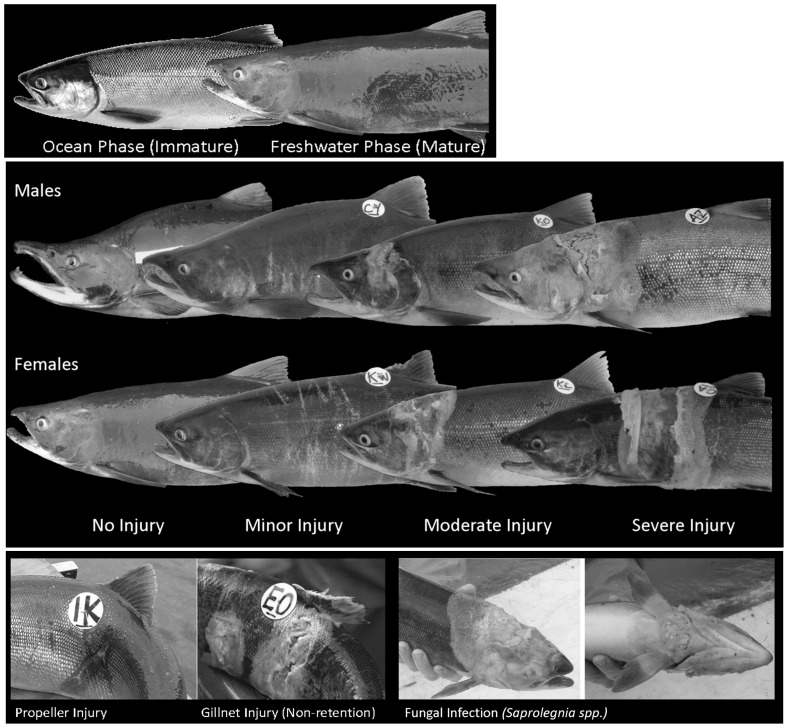
Top Panel: Sockeye salmon at in-river migration, (early-stage maturation, top left) and at spawning streams (late-stage maturation, top right). Middle Panel: Sockeye salmon categorized according to severity of non-retention injury as sampled at spawning streams. Traits associated with sexual maturity, such as darker coloration (both sexes), absorbed scales (both sexes), dorsal-ventral elongation (males), and increased kype length (males) are typically reduced as a function of increased severity of disentanglement injury. Bottom Panel: Propeller injuries were characterized by a clean cut with limited extent in contrast to gillnet disentanglement injuries, characterized by multiple points of contact with lacerations spanning the circumference of the fish (bottom left). Fungal infection, *Saprolegnia spp.* (bottom right).

Non-retention (here defined as active or passive disentanglement from capture gear, rather than deliberate release by humans) is prevalent in commercial fisheries, but there is limited knowledge of the consequences to escaped fish and impacts of unaccounted mortality [1], [2]. Physiological analyses provide an opportunity to examine mechanisms [3], which cause escape mortality. Salmon (*Oncorhynchus spp.*) stocks in Alaska are managed to ensure sustainable production by allowing a fixed number of adult fish to escape commercial fisheries and return to natal sites to spawn [4], [5]. In stocks subject to harvest with commercial gillnets, many fish that ultimately escape may temporarily entangle in nets while migrating through fishing districts (Fig. 1). Non-retention in gillnet fisheries leads to disentanglement injuries in escaped fish and may degrade the reproductive potential of spawning stocks. Previous studies [6], [7] have demonstrated that such injuries have detrimental effects, delaying sexual maturation and timing of spawning, and often result in pre-spawning mortality. In this study we reveal the mechanisms by which delayed mortality and spawning failure might occur.

### Elevated stress related to non-retention in gillnet fisheries

Across a range of fisheries and harvest strategies, physical injuries to fish from interactions with fishing gear include fatigue and physiological stress, external and internal injuries, suffocation, and barotrauma [2], [8], reviewed in Chopin & Arimoto [9]. While few studies have analyzed effects of disentanglement from gillnets in wild stocks, several studies have analyzed simulated effects of capture and entanglement in controlled studies. Injuries specific to gillnet entanglement include compression, internal injuries, abrasions, and disruption of the mucous layer, which increases susceptibility to pathogens. Constriction in gillnets may induce fatal damage to the circulatory system [10] and extreme exertion related to disentanglement may contribute to respiratory acidosis [11]. Severity and extent of injuries increase with time of entrapment [12]. Both immediate and delayed mortality subsequent to disentanglement is likely to occur with increasing severity of injury [6], [9], [12], [13]. Prolonged entanglement or multiple interactions with acute stressors may lead to chronic physiological stress [14], which may alter behavior [15], lower immune responses [16], impair physiological performance (e.g. osmoregulation [17]), and in extreme instances, inhibit sexual maturation and reproduction [18], [19], [20]. Chronic physiological stress associated with non-retention or related injuries may lead to delayed mortality [21], such that, absent physiological analyses, a distressed condition may be indiscernible for a considerable period of time after the initial stressor [22].

### Stress response in fishes

The stress response is a regulatory response mechanism to return to homeostatic norms [20]. Sustained stress, however, has detrimental effects in fish and may inhibit processes such as growth, reproduction and disease resistance [14]. Stress occurs in a sequence of (i) alarm; (ii) resistance, and (iii) exhaustion stages. The primary physiological response in the initial alarm phase is the rapid secretion of catecholamines and corticosteroid hormones. These initiate a sequence of secondary biochemical responses [23], which mobilize energy reserves, enabling the fish to resist stressors. Chronic exposure to these conditions will lead to exhaustion, degraded physical condition, and ultimately death [23], [24]. Cortisol is the major glucocorticoid in salmonids [25] and is commonly used as an indicator of chronic stress [26]. While capture and handling may elevate cortisol levels, cortisol synthesis and release has a lag time of several minutes after exposure to a stressor [27]. A well-designed sampling protocol will therefore enable the measurement of baseline levels [24].

### Cortisol, immunodeficiency, and reproduction

There is extensive documentation on the reduced immunological capacities of fish experiencing acute [28] and chronic [29] exposure to stress. Increased stress and elevated cortisol may have metabolic costs [18], lead to compromised immune function (reviewed in Ellis [30]), and reduce resistance to pathogens (e.g. increase susceptibility to freshwater molds (*Saprolegnia spp.)* in salmonids, [29]).

There is also substantial evidence that physiological stress has inhibitory effects on reproduction in teleost fishes as the result of modified endocrine function (reviewed in Pankhurst & Van Der Kraak [18]. Repeated exposure to acute stress and prolonged exposure to chronic stressors disrupt endocrine processes associated with reproduction in salmonids, leading to ovarian atresia and reduced reproductive success [31], [32], [19].

At the same time, cortisol concentrations must be interpreted in the context of other physiological and environmental processes [33]. In the case of returning Pacific salmon, hypercortisolemia may result from several factors, including changes in environment and salinity, demands of migration, gonadal development, and starvation [34]. Plasma cortisol levels in adult salmonids increase in association with migration and homing, reproductive maturation, osmoregulatory and metabolic challenges related to transition from marine to freshwater environments, and the onset of senescence [21], [35].

Sexual maturation in salmonids is associated with a variety of morphological, physiological and behavioral changes largely influenced by circulating androgens [36]. At maturity sockeye salmon (*Oncorhynchus nerka*) exhibit striking coloration differences from the immature ocean phase (Fig. 1), as well as sexual dimorphism. Females retain the fusiform shape. Males, in contrast, become laterally compressed, developing a prominent hump anterior to the dorsal fin. The kype or upper jaw elongates and prominent teeth emerge. Both sexes develop distinctive coloration, flushing carotenoid and lipophilic pigments from muscle tissue to the skin, shifting from silver coloration to deep red [36], [37]. Skin thickens and scales are resorbed [38]. Thus both coloration and morphology serve as important indicators of sexual maturity and may be used to highlight delayed or inhibited reproduction, particularly as secondary sexual characteristics in sockeye salmon develop after entry into freshwater and passage through the fishery [39].

### Sex steroid profiles for reproductive maturation in salmon

Sexual maturation in salmonids is primarily regulated by the pituitary gonadotropins (GTHs), follicle-stimulating hormone (FSH) and lutenizing hormone (LH), which promote the synthesis of gonadal sex steroid hormones such as testosterone (T), 11-ketotestosterone (11KT), estradiol-17β (E2) and 17,20β-dihydroxy-4-pregnen-3-one (17,20β-DHP) [40]. There is a clear and well-defined profile of sexual steroids related to the process of sexual maturation in female Pacific salmon (Fig. S1). Plasma concentrations of E2, which promotes hepatic vitellogenin synthesis, increase during the months preceding spawning and typically peak during river migration. E2 levels then decline rapidly just prior to final oocyte maturation, ovulation, and spawning (reviewed by Nagahama & Yamashita [41]). In contrast, concentrations of (17,20β-DHP), which promote final oocyte maturation are low throughout vitellogenesis but rapidly increase just prior to ovulation and spawning, and remain elevated throughout the duration of spawning activity [41], [42] and to senescence [43], [44]. 17,20β-DHP is produced by salmonids immediately prior to spawning, regulates final maturation in both sexes [45] and has been shown to trigger final maturation *in vitro* [46] and *in vivo* [47].

Due to the unique and complementary roles and profiles for E2 and 17,20β-DHP, their relative values may be used in concert to evaluate maturation in individual adult females immediately prior to spawning, such that: (i) high E2 and low (17,20β-DHP) indicate ongoing maturation (development of gametes); (ii) low E2 and high (17,20β-DHP) indicate full maturation; and (iii) low levels of both steroids indicate delayed or possibly inhibited maturation.

### Approach

Our analyses examined physiological stress (via plasma cortisol) and sex steroid profiles (E2, 17,20β-DHP) in sockeye salmon that disentangled in commercial gillnet fisheries to better understand mechanisms for reproductive failure and pre-spawning mortality. Relative to uninjured fish, we anticipated: (i) elevated physiological stress as evidenced by elevated cortisol; (ii) delayed or inhibited development of secondary sexual morphological traits; and (iii) altered reproductive physiology that would suggest a delayed maturation schedule or failure to mature.

## Material and methods

### Blood sampling protocol

Sockeye salmon were sampled at two stages: (i) in-river migration (early-stage maturation); and (ii) aggregations at the mouth of spawning stream (late-stage maturation) in years 2006–2008. In-river sampling occurred in the Wood River, Bristol Bay Alaska (59°15′05″N 158°34′32″W, *n* = 115) approximate to the peak of the run (July 5–10). Sampling at spawning streams occurred at 10 stream sites (*n* = 2365) within the Wood River system in accordance with historical peak spawning date (late July to early August). Water temperature at stream sites ranged 8–11°C (Mean = 9.4±0.3 SE). The distance between the fishery and in-river sampling site is 50 km. Residence time within the fishing district is approximately 48 hours and transit time between the fishing district and in-river sampling site is 12–48 hours (Tim Sands, Alaska Department of Fish and Game, personal communication). All fish sampled were surveyed for coloration, presence and severity of injury, presence of fungal infection, and morphology; subsamples representing a range of injury severity were processed for analyses of cortisol (*n* = 204) and reproductive hormones (*n* = 102). To provide a uniform metric, coloration was determined on the basis of scale resorption, such that fish with complete scales were ‘silver’, partial resorption were ‘blush’ and full resorption were ‘red’. Fish were categorized according to severity of injury as described by Baker & Schindler [6] (Fig. 1).

Immediately upon capture by beach seine, fish were immersed in a buffered 300 ng/mL solution of tricaine methanesulfonate (MS-222) to induce respiratory failure. High concentrations of MS-222 are recommended for prevention of stress-related changes in blood chemistry [48] and have no effect on plasma cortisol levels in salmonids [49]. Blood was collected from the caudal vasculature into heparinized tubes. After centrifugation, plasma was stored at −80°C until analysis. Subsequent to blood extraction, fish were measured for length (mid-eye to hypural plate), depth (immediately anterior to dorsal fin) and kype (mid-eye to tip of snout). Otoliths were extracted to determine age. Coloration, presence of fungal infection (likely *Saprolegnia spp.*) and severity of gillnet disentanglement injuries were noted (Fig. 1).

### Controlling for capture and handling stress during sampling

Due to latency in the cortisol response to an acute stressor, it is possible to measure baseline levels within a narrow timeframe of initial disturbance [48]. Mean response latency for a measurable increase in plasma cortisol in teleost fishes is 12.5 minutes (reviewed in Pankhurst [50]). A number of studies have also shown that euthanasia with overdose of MS-222 within a 15-minute timeframe provides reliable values for baseline levels [51], [52]. All fish analyzed in this study were sampled in <12.5 minutes (i.e. initial disturbance caused by the initiation of the beach seine to deposition in a lethal anesthetic bath and blood extraction). Mean sampling time was 5.92±1.79 (SD) minutes for in-river sampling (migrating stage) and 7.1±1.4 minutes at spawning streams (late-stage maturation).

### Cortisol assays

Cortisol concentrations in plasma were measured using a double antibody radioimmunoassay kit ( DSL-2000) from Diagnostic System Laboratories, Inc., Webster, TX. Dilutions of plasma produced a curve parallel to the standard curve. Inter-assay variation was 6.4%. Intra-assay variation was 2.7%.

### Sex steroid assays

Analyses were conducted at the Environmental Physiology Program at the Northwest Fisheries Science Center, National Oceanic and Atmospheric Administration, Seattle, WA. Steroids were extracted from plasma using a double extraction with diethylether (1.5 mL ether/0.175–0.2 mL plasma). Extraction efficiencies in assays conducted at the Environmental Physiology Program are regularly evaluated and consistently greater than 90%. No corrections of the data for efficiency were made. Extracts were dried at 37 C under nitrogen atmosphere, re-suspended in phosphate buffered saline containing 0.2% gelatin. Estradiol was measured in female plasma samples via radioimmunoassay (described by Sower & Schreck [53]) by using an antibody (cross-reactivities: estrone, 2.6%; estriol, 4.2%; testosterone, 0.02%; [54]) purchased from G. Niswender (Colorado State University, Fort Collins). The radioimmunoassay for 17, 20β-DHP was performed according to Planas et al. [55], using an anstisera and protocols provided by A.P. Scott [56] (cross-reactivities: progesterone, 0.002%; 17a-hydroxyprogesterone, 0.015%; cortisol, 0.05%; estradiol, <0.002%; testosterone <0.002%). Inter-assay coefficients of variation were 5.89% and 9.85% for E2 and DHP, respectively.

### Statistical analyses

To account for non-normality and heterogeneity in the data, we applied hierarchical and nonparametric approaches to evaluate differences in cortisol and reproductive hormones as a function of sex, disentanglement injury, and fungal infection. For each stage (river, streams), differences in cortisol and sex steroids (E2, 17,20β-DHP) were evaluated via hierarchical approaches to account for the possibility of correlated error within groups and to facilitate interpretation of predictor variables. To determine relative effects and account for all potential interactions between sex, presence of fungal infection, and severity of injury, we adopted mixed effects models that incorporated sex, injury and fungal infection as fixed effects and year (and stream, for stream stage analyses) as random effects. To determine the optimal model given explanatory variables and their interactions, we compared nested models and sequentially dropped non-significant interaction terms. Sex steroids were evaluated in females only. Differences in cortisol values between sexes are typical in physiological analyses of stress and were anticipated; therefore all values are reported separately for each sex. Wilcoxon (Mann-Whitney) rank tests were applied to evaluate differences in cortisol values and sex steroids on the basis of maturation stage (river versus streams, uninjured fish). Kruskal-Wallis tests were applied to determine differences in cortisol according to coloration (at streams). Nonparametric multiple comparison tests (R package pgirmess, kruskalmc) were applied to evaluate pairwise comparisons (analogous to Tukey post-hoc tests) when significant differences were found. ANCOVA was used to evaluate reductions in depth and kype length according to severity of injury (streams, males), with body length as a covariate.

Research protocols were approved by the Committee on Animal Care at the University of Washington Experiments of the University of Minnesota (Permit Number: 3142-01). All efforts were made to minimize suffering. All necessary permits were obtained through the Alaska Department of Fish and Game (Permit Number SF2008-090).

## Results

### Morphological traits and coloration

**Figure 2:**
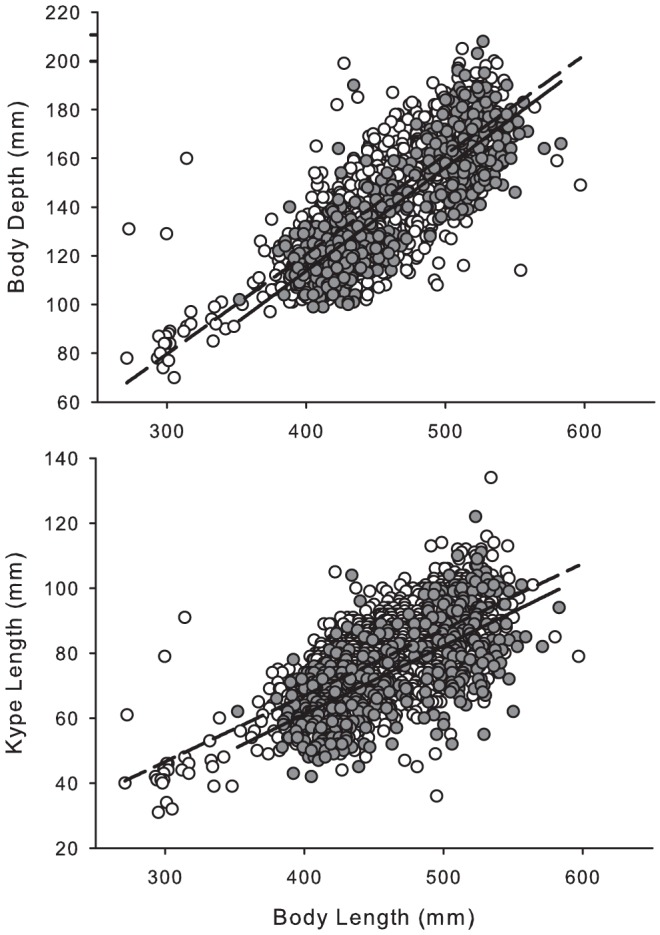
Linear regression of body depth (top panel) and kype length (bottom panel) as a function of body length in males sampled at spawning streams. Uninjured fish (○,– –, Mean Length 452±0.97 mm SE, *n* = 1864) exhibited increased traits associated with reproduction maturity relative to injured fish (•, _ , Mean Length 462±2.18 mm SE, *n* = 501).

**Figure 3:**
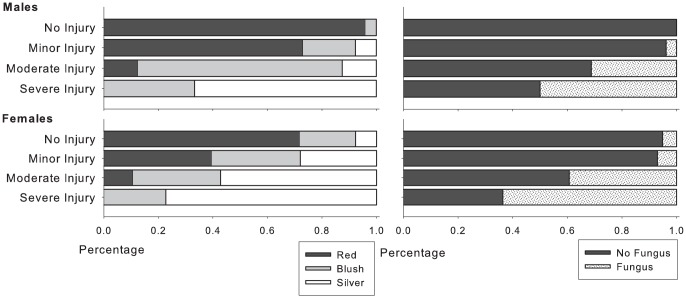
Coloration (left panel) and prevalence of fungal infection (right panel) at spawning streams, as a function of severity of gillnet injury (*n* = 2365). As color is a qualification of a gradient characteristic, fish were also evaluated as per the extent to which scales were resorbed to further standardize color ranking.

Injuries sustained from non-retention in gillnets inhibited development of secondary sexual characteristics associated with maturation in sockeye salmon. Among males sampled at spawning streams, both body depth and kype length were reduced as a function of body length in injured fish relative to uninjured fish (ANCOVA: *F* = 123.89, *P* = 0.000, Fig. 2). The reduction of male secondary sexual characteristics scaled with severity of disentanglement injury (ANCOVA, depth: *F*_2,498_ = 23.55, *P* = 0.000; kype: *F*_2,498_ = 21.54, *P* = 0.000). Additionally, coloration associated with sexual maturation was reduced in both males and females injured via non-retention (Fig. 3).

### Indicators of chronic stress: cortisol, disentanglement injuries, and fungal infection

As expected, we found significant differences in cortisol levels between the sexes (uninjured fish) in-river (migrating, Mann-Whitney, *U*_14,40_ = 368, *P* = 0.041) and at spawning streams (late-stage maturation, Mann-Whitney, *U*_25,52_ = 1079, *P* = 0.000), such that females were elevated 200–350% relative to males.

**Figure 4:**
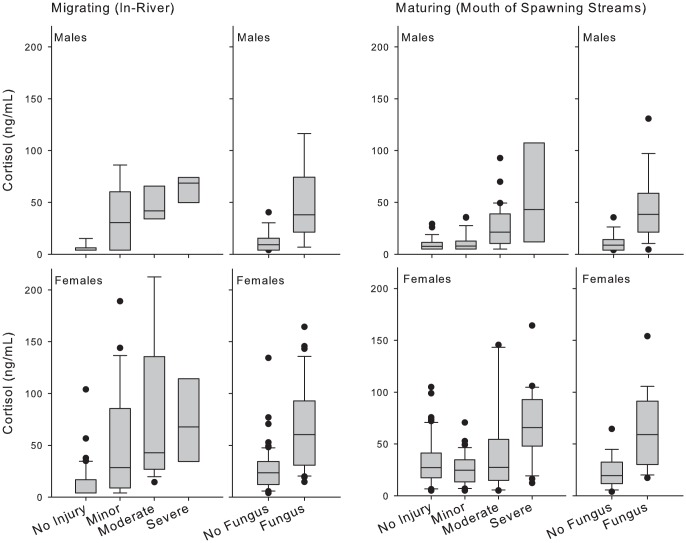
Plasma cortisol (ng/mL) in sockeye salmon sampled during in-river migration or at spawning stream mouths, according to severity of gillnet injury and presence of fungal infection (*Saprolegnia spp*.). Boxes mark the 25th–75th percentiles, the line marks the median, and whiskers mark 90th and 10th percentiles.

We found that cortisol levels increased in accordance with presence and severity of injury both in-river and at spawning streams (Table 1, Fig. 4). In analyses of cortisol in-river, linear mixed effects (lme) models indicated significant differences between sexes (*t*_2,109_ = 1.82, *P* = 0.043) and between injured and uninjured fish (*t*_3,109_ = 3.47, *P* = 0.001), but not between discrete levels of injury. No significant interactions were noted between sex, severity of injury, and fungal infection. In analyses of cortisol at streams, lme models indicated significant differences between sexes (*t*_2,198_ = 4.06, *P* = 0.000) and between all categories of injury (*P*<0.001), with the exception of fish with minor injuries and uninjured fish (*t*_3,198_ = 0.99, *P* = 0.3255). Significant interactions were noted between severe injuries and fungal infection (*t*_2,198_ = 2.07, *P* = 0.039), but no other level of injury. No significant interactions were noted between sex and either severity of injury or fungal infection. Fungal infection was often associated with disentanglement injuries (Fig. 3); significant differences in cortisol levels were noted on the basis of fungal infection in-river (*t*_2,198_ = 4.79, *P* = 0.000), but not at streams (*t*_2,198_ = 0.13, *P* = 0.895).

**Table 1:** Cortisol in salmon by location (ng/mL), mean (SE).

	Severity of Disentanglement Injury				Presence of Fungal Infection	
Females						
Reproductive Stage	No Injury	Minor Injury	Moderate Injury	Severe Injury	No Fungus	Fungus
In-River Migration	14 (3.0), *n* = 40	48 (11.3), *n* = 21	77 (19.6), *n* = 15	73 (18.8), *n* = 5	23 (3.3), *n* = 64	95 (18.7), *n* = 17
Spawning Stream Mouth	33 (3.2), *n* = 52	26 (2.1), *n* = 45	47 (17.1), *n* = 10	68 (7.2), *n* = 22	29 (3.0), *n* = 98	63 (6.8), *n* = 30

Males						
Reproductive Stage	No Injury	Minor Injury	Moderate Injury	Severe Injury	No Fungus	Fungus
In-River Migration	7 (1.1), *n* = 14	33 (8.6), *n* = 13	47 (9.0), *n* = 4	64 (7.5), *n* = 3	22 (4.7), *n* = 30	60 (6.5), *n* = 4
Spawning Stream Mouth	9 (1.2), *n* = 25	11 (1.8), *n* = 26	27 (3.7), *n* = 32	57 (21.2), *n* = 6	14 (1.5), *n* = 75	49 (9.9), *n* = 14

Notes: Cortisol levels in migrating and maturing salmon. All sampled fish (regardless of presence or severity of gillnet disentanglement injuries) were included in estimates for fish with/without fungal infection. Cortisol levels increased in accordance with presence and severity of injury; Nonparametric multiple comparison post-hoc tests confirmed significant differences (*P*<0.001) between uninjured fish and all categories of injury at in-river migration, and in all pair-wise comparisons at streams except uninjured-minor fish.

### Cortisol and coloration associated with sexual maturation

In uninjured fish (in-river and spawning streams combined), cortisol levels increased with coloration associated with reproductive maturity (Kruskal-Wallis, uninjured females: χ^2^_2,77_ = 29.53, *P* = 0.000; uninjured males: *F*_2,35_ = 12.78, *P* = 0.002).

### Reproductive hormones

**Figure 5:**
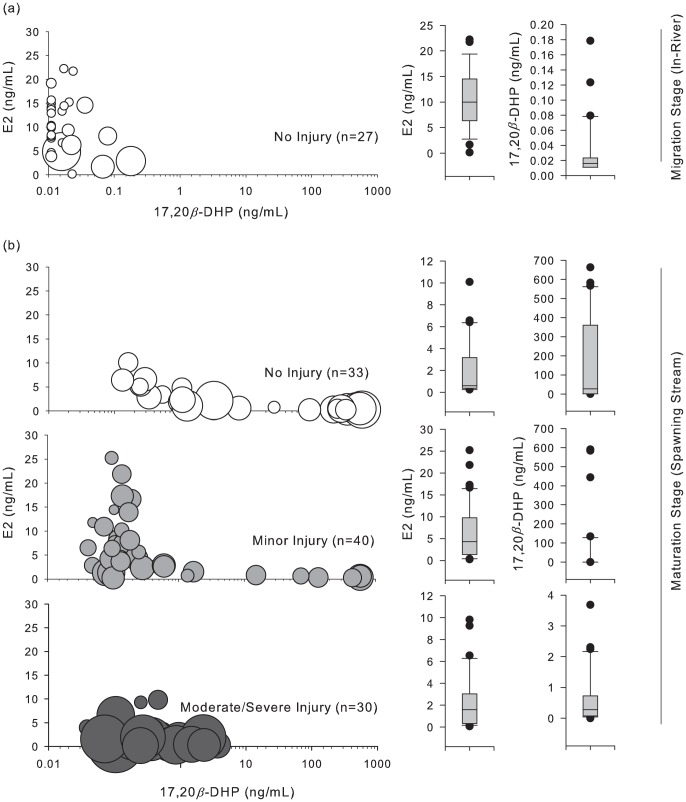
Profile of plasma sex steroids (E2, 17,20β-DHP) in female sockeye salmon. Profiles at in-river migration are displayed for uninjured fish to provide a reference for the natural progression of sex steroids at migration and maturation stages (top panel, open circles). Profiles at spawning streams are displayed according to severity of disentanglement injury, including uninjured fish (open circles), fish with minor injuries (light gray circles), and fish with moderate-to-severe injuries (dark gray circles). The symbol area represents the relative value of plasma cortisol, such that large symbols reflect elevated cortisol.

As expected, we observed marked differences in sex steroid profiles for females (uninjured) at in-river migration compared to at spawning streams (Mann-Whitney, E2: *U*_26,33_ = 782, *P* = 0.000; 17,20β-DHP: *U*_26,33_ = 0, *P* = 0.000, Fig. 5). In uninjured females, E2 was elevated in-river (10.9±1.1 SE ng/mL) and subsequently declined at spawning streams (2.1±0.4 ng/mL), whereas 17,20β-DHP was negligible during in-river migration (0.01±0.0 ng/mL, non-detectable values were interpreted as zero) and increased exponentially in most fish at spawning streams (190.5±39.1 ng/mL). At spawning streams, 55% (*n* = 18 of 33) of uninjured females had levels of 17,20β-DHP indicative of the onset or completion of ovulation (>10 ng/mL). On the basis of dissection and analysis of the skein and egg cavity, these fish were considered mature and reproductively viable. In contrast, only 15% (*n* = 6 of 40) of females with minor injuries and none (*n* = 0 of 30) with moderate or severe injuries had 17,20β-DHP elevated to those levels (Table 2, Fig. 5). Females with minor injuries at spawning streams had E2 (6.4±1.0 ng/mL) elevated to levels proximate to E2 levels recorded in uninjured fish at migration (10.9±1.1 ng/mL); 17,20β-DHP levels, however, remained relatively low (46.2±23.0 ng/mL; >1.0 ng/mL in 80% of fish), suggesting delayed or inhibited maturation. In females with moderate and severe injuries at spawning streams, both E2 (2.3±0.5 ng/mL) and 17,20β-DHP (0.6±0.1 ng/mL) were depressed, suggesting delayed or inhibited sexual maturation. Analysis of the skein and egg cavity confirmed these results (eggs tightly retained within the skein, rather than loose within the skein or body cavity). This suggested that disentanglement from commercial gillnet fisheries delayed and possibly inhibited maturation, therefore reducing reproductive performance and success.

**Table 2:** Sex steroid levels in females at spawning streams (late-stage maturation) (ng/mL, mean ± SE).

	Severity of Disentanglement Injury			Presence of Infection *(Saprolegnia spp.)*		Coloration		
Sex Steroids	No Injury	Minor Injury	Moderate or Severe Injury	No Fungus	Fungus	Red	Blush	Silver
Estradiol	2.1 (0.4), *n* = 33	6.4 (1.0), *n* = 40	2.3 (0.5), *n* = 32	4.5 (0.6), *n* = 78	1.6 (0.3), *n* = 25	2.4 (0.5), *n* = 44	6.6 (1.2), *n* = 26	3.4 (0.7), *n* = 32
17,20β-DHP	190.5 (39.1), *n* = 33	46.2 (22.9), *n* = 40	0.6 (0.1), *n* = 32	102.6 (21.9), *n* = 78	6.2 (5.4), *n* = 25	184 (34.4), *n* = 44	0.7 (0.3), *n* = 26	0.4 (0.1), *n* = 32
Percent Mature	55%	15%	0%	32%	4%	52%	0%	0%

Notes: Sex steroid levels for females at spawning streams. Females with 17,20β-DHP levels >10 ng/mL were determined to be mature, indicating that ovulation had either initiated or occurred. All fish (regardless of presence or severity of gillnet disentanglement injuries) were included in estimates for fish with/without fungal infection and coloration. In evaluating relative effects of injury and infection on reproductive hormones, severity of injury was a better predictor than fungal infection for E2 (Kruskal-Wallis: Injury: *χ^2^*_2,102_ = 19.5, *P* = 0.000; Infection: *χ^2^*_1,102_ = 8.6, *P* = 0.041) as well as 17,20β-DHP (Kruskal-Wallis: Injury: *χ^2^*_2,102_ = 27.2, *P* = 0.000; Infection: *χ^2^*_1,102_ = 0.9, *P* = 0.342).

**Figure 6:**
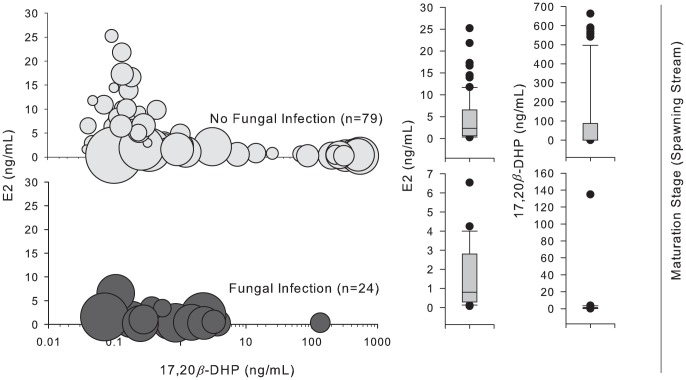
Profile of plasma sex steroids (E2, 17,20β-DHP) in female sockeye salmon. Profiles at spawning streams are displayed according to presence (dark gray circles) or absence (light gray circles) of fungal infection, regardless of the presence or severity of disentanglement injury.

In analyses of E2 (streams), lme models indicated significant differences for uninjured fish (*t*_3,102_ = 2.98, *P* = 0.004). In analyses of 17,20β-DHP (streams), lme models indicated significant differences between all categories of injury (*P*<0.020). No significant effects or interactions were noted with regard to fungal infection. Nearly all females with fungal infection had low levels of both E2 and 17,20β-DHP, suggesting reproductive failure. Many with gillnet injuries, but without fungal infection, also displayed low levels of both steroids, however, suggesting that fungal infection, while correlated with inhibited reproduction, is not necessarily the proximate cause (Table 2; Fig. 6).

## Discussion

### Evidence that non-retention inhibits sexual maturation and reduces reproductive potential

We found evidence for delayed and/or inhibited sexual maturation in sockeye salmon that disentangle from commercial gillnets and migrate to freshwater spawning habitats. Both a delay in the development of secondary sexual morphological traits and a delay in reproductive physiology were evident, providing further insight as to the physiological mechanisms by which non-retention might elevate pre-spawning mortality and degrade reproductive potential in spawning stocks.

### Inhibited morphological development associated with sexual maturity

The development of secondary sexual characteristics in salmon occurs after passage through commercial fisheries and entry into freshwater. This morphological transformation constitutes a high energetic cost on limited endogenous resources [39]. Given the energy expended in the struggle to escape entanglement, recover from injury and resist infection, injured fish may not retain sufficient energy reserves necessary to complete sexual maturation [57], [58]. Male salmon with severely diminished energy reserves at maturation have been shown to have reduced body depth and kype length [59]. Alternatively, elevated cortisol levels related to injuries may suppress androgen production [19] and expression [60], reducing the prominence of these secondary sexual traits. Further research should investigate the potential for reduced androgen levels in males and reduced energy reserves overall. Reduced energy reserves may reflect a diminished capacity to compete for habitat and mates [39], reduced longevity on spawning grounds [61], [62], and may induce aberrant behavior [63] – all factors that negatively affect spawning success.

### Fungal infection

Fungal infection is an important indicator for degraded condition and reproductive failure (Baker and Schindler [6]) and may be a proximate cause of mortality. It remains unclear whether infection is prevalent in injured fish because conditions for colonization are enhanced by dermal abrasions from disentanglement injuries or whether it is facilitated by chronic stress. S*aprolegnia spp.* and other fungal pathogens are a threat to spawning salmon generally. Salmon are more susceptible to infection during transition to fresh water and while in dense spawning aggregations [64]. Mark-recapture studies [6] found that fish with fungal infections fail to mature and hold in dense aggregations for extended periods. This may increase opportunities for transmission of pathogens, elevate infection rates in uninjured fish, and represent an additional indirect mechanism by which non-retention in fisheries compromises reproductive health of spawning stocks.

### Cortisol and chronic stress: critical thresholds

There appears to be a critical stage at which salmonids are most susceptible to the inhibitory effects of stress on reproductive maturation. Effects are more marked in immature stages [65] and salmon are less sensitive to external stressors at advanced stages of sexual maturity [65]; there is considerable evidence for attenuated corticosteroid responses to acute and chronic stressors [19], [66], [67], and increased metabolic clearance rates of cortisol [65], [66] in mature salmonids. Our results counter theoretical predictions that suggest semelparous animals should mute glucocorticoid stress responses to focus on reproductive processes [68], [69]. Whereas experimental manipulations on fully mature spawning fish suggest a muted stress response [70], our results indicate that external stressors experienced prior to maturation delay or inhibit maturation. This distinction is critical, as salmon typically experience stressors related to commercial fisheries at an immature state. Given levels observed, cortisol is likely not a proximate driver of reproductive failure. However, elevated cortisol in the absence of elevated sex steroids serves as an important indicator of compromised physiology and degraded condition.

### Interpreting causation in the relationship between cortisol and sexual maturation

The inhibitory effects of stressors on reproductive performance have been demonstrated for several teleost species [26]. Chronically elevated plasma cortisol levels may decrease androgen and estrogen production [65], [71], suppress reproductive development and maturation and impair gonadal steroidogenesis [31] (but also see Pankhurst [72]), delay ovulation [19], [32], impair gamete quality [73] and larval viability [32], and correlate with pre-spawning mortality [74].

Elevated cortisol in salmonids, however, may also reflect processes associated with reproductive maturity rather than indicate chronic stress. In female salmonids, cortisol levels have a regulatory role in final oocyte maturation and ovulation [47] and spawning [75]. A close temporal relationship between plasma profiles of cortisol and 17,20β-DHP in reproductively mature salmonids suggests 17,20β-DHP directly contributes to periovulatory hypercortisolism [76]. Cortisol has been shown to reach extremely high levels in spawning salmon [19] and stress responsiveness decreases with sexual maturation [25], suggesting that salmon are less responsive to cortisol at maturity. Thus responses to stressors may be difficult to distinguish from maturation and reproductive processes [33].

In the case of commercially exploited salmon in Alaska, final maturation occurs after exposure to non-retention in commercial fisheries. Therefore, while elevated cortisol at later maturation stages may be driven by processes associated with sexual maturation, elevated cortisol at migration is indicative of increased physiological stress, with associated deleterious effects on fitness. In our study, uninjured fish were characterized by low cortisol at migration and elevated cortisol and elevated 17,20β-DHP at spawning streams. In contrast, fish with injuries were characterized by elevated cortisol at both stages and low levels of 17,20β-DHP at spawning streams. This suggests that elevated cortisol in fish injured via entanglement in commercial fisheries is indicative of chronic physiological stress.

### Sex steroid profiles and mechanisms for delayed and inhibited maturation

Multiple mechanisms driving cortisol production and confounding relationships with cortisol, androgens and estrogens complicate efforts to interpret cortisol in maturing and reproductively active salmonids. Sex steroids may be more informative and definitive as biomarkers for reproductive potential than cortisol. 17,20β-DHP regulates final maturation in salmonids and is therefore a sensitive indicator of reproductive maturity [45], [46]. In concert, analyses of E2 and 17,20β-DHP provide insight into reproductive maturation [40]. At stages where maturity is expected (spawning streams), highly elevated DHP indicates reproductive maturity, whereas declining E2 with rising 17,20β-DHP indicates ongoing reproductive maturation. In contrast, highly elevated E2 and low 17,20β-DHP suggest delayed maturation; low levels of both suggest critically delayed or inhibited maturation.

In fish with moderate and severe injuries, the failure to produce 17,20β-DHP provides compelling evidence of reduced reproductive potential, especially as E2 levels are low and cortisol levels high. In fish with minor injuries, delayed or inhibited 17,20β-DHP production in concert with elevated E2, may indicate delayed reproductive maturation. While these results might arise from delayed arrival (i.e. extended residence in the river as a result of encounters with fishing gear), a delay would itself suggest diminished reproductive potential. Factors affecting ovarian development that delay maturation likely also reduce reproductive success. Gonadal maturation and spawning are largely under the control of biochemical mechanisms, which impose a relatively inflexible schedule [77]. Moreover, migratory and maturation schedules for salmon are constrained within a narrow timeframe, such that deviations from that schedule may adversely impact spawning success [43]. Given this evidence, the delay in reproductive maturation evident in fish with minor injuries is strongly suggestive of compromised reproductive performance. Future research should examine this directly.

### Implications for management

We provide physical and physiological evidence that reproductive maturation is delayed or inhibited in salmon injured by non-retention in commercial fisheries. Our measurements of sex steroids in maturing fish suggest disentanglement injuries delay or inhibit reproductive maturation and reduce reproductive fitness. These findings are further supported by the absence of morphological traits associated with sexual maturation (coloration, kype development, and dorsal-ventral elongation) in disentangled fish. The underlying drivers likely include not only physical injury, but also physiological stress and pathogen exposure. Our results further understanding of the mechanisms by which non-retention leads to reproductive failure, and thus pre-spawning mortality (as evident in Baker & Schindler [6]).

Pre-spawning mortality due to non-retention in commercial fisheries has not prevented sustainable harvests of salmon stocks in Alaska. Target spawning escapement ranges are determined on the basis of trial and error and past performance [78], and therefore implicitly account for reproductive failure of fish enumerated in the escapement. But explicitly quantifying, understanding, and accounting for this mortality would improve management and might enable increased yield. Related research [79] has shown that non-retention correlates with fishing effort and that escape mortality reflects the exploitation rate on escaped stocks. This suggests indices of fishing effort may be used to predict such mortality and improve estimates of the number of effective spawning fish. Understanding the physiological mechanisms leading to mortality following disentanglement will enable managers to better understand the impact of escape mortality on spawning stocks. Given the prevalence of injuries in spawning stocks, it is worth considering how management might reduce the incidence and severity of gillnet disentanglement injuries. Targeted temporal closures may be useful in the context of a line fishery to reduce incentives for long soak times during periods of low activity. This might reduce the likelihood of disentanglement overall and also reduce severity of injury in those fish that disentangle, since fish that spend longer durations entangled prior to escape incur more severe injuries [80].

## Supporting Information

Figure S1 **Spline curves fit to plasma concentrations of E2 versus 17,20β-DHP as a function of advancing ovarian maturation (top graph) or migration stage (bottom graph ) for coho (** ***Oncorhynchus kisutch*** **) and masu samon (** ***O. masou*** **).** Reproductive maturation stage profiles were reconstructed from multiple year studies (year = 2) (Fitzpatrick et al. 1986). Migration stage profiles reconstructed from multiple year study (Onuma et al., 2003), and studies by Onuma et al. (2009) and Ueda et al. (1998).
(TIF)

File S1 **Supplementary Text: reproductive steroid profiles in maturing female salmon.**
(DOCX)
