# Supplementary figures and images for: Injuries from Non-Retention in Gillnet Fisheries Suppress Reproductive Maturation in Escaped Fish
Source: PLoS One. 2013 Jul 24;8(7):e69615. doi: 10.1371/journal.pone.0069615 (PMC3722223; doi:10.1371/journal.pone.0069615)

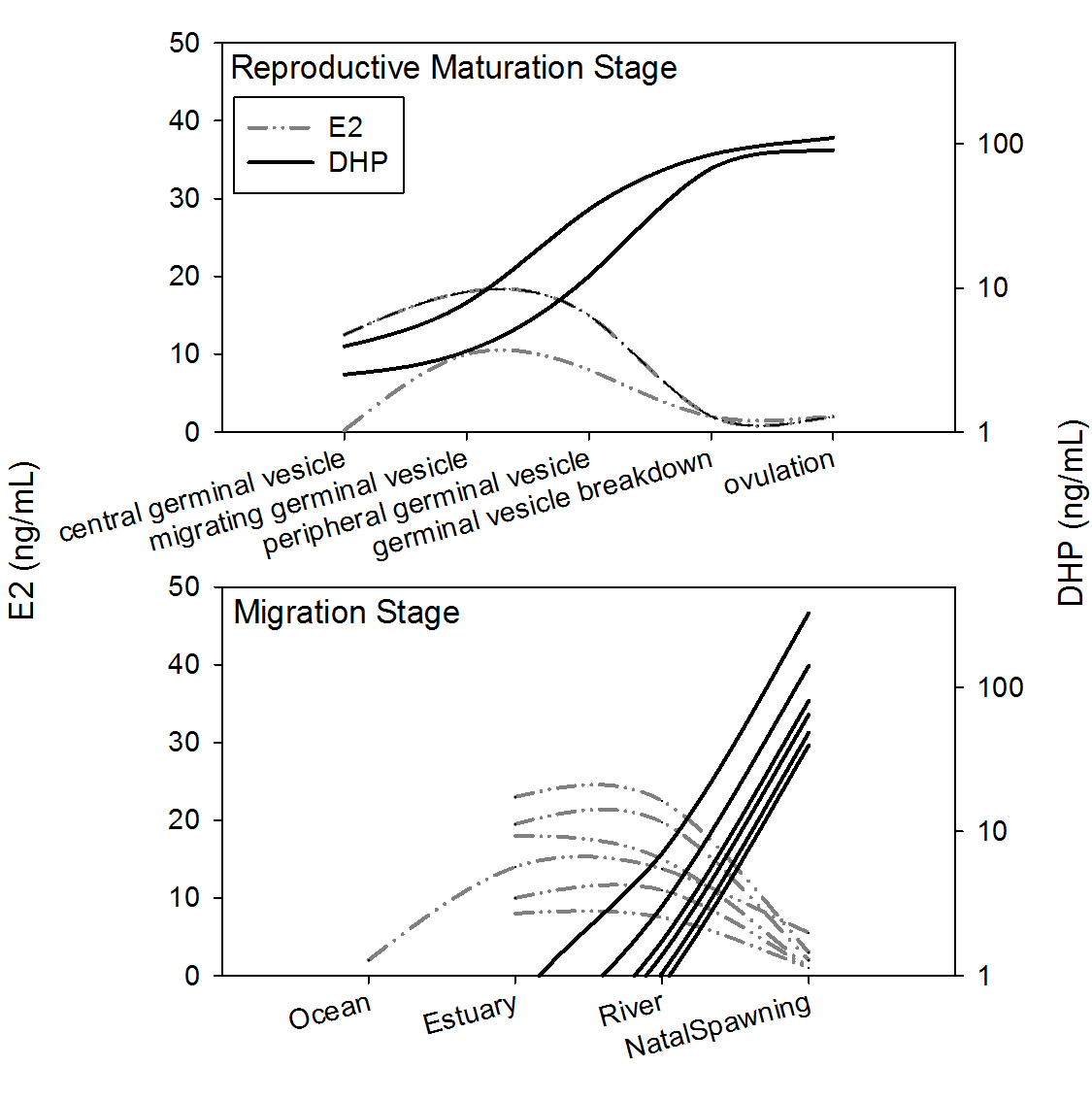

Supplement: Figure S1 — Spline curves fit to plasma concentrations of E2 versus 17,20β-DHP as a function of advancing ovarian maturation (top graph) or migration stage (bottom graph ) for coho ( Oncorhynchus kisutch ) and masu samon ( O. masou ). Reproductive maturation stage profiles were reconstructed from multiple year studies (year = 2) (Fitzpatrick et al. 1986). Migration stage profiles reconstructed from multiple year study (Onuma et al., 2003), and studies by Onuma et al. (2009) and Ueda et al. (1998). (TIF) [file pone.0069615.s001.tif]
