# Supplementary material for: Injuries from Non-Retention in Gillnet Fisheries Suppress Reproductive Maturation in Escaped Fish
Source: PLoS One. 2013 Jul 24;8(7):e69615. doi: 10.1371/journal.pone.0069615 (PMC3722223; doi:10.1371/journal.pone.0069615)
Supplement: File S1 — Supplementary Text: reproductive steroid profiles in maturing female salmon. (DOCX) [file pone.0069615.s002.docx]

**Supporting Information: Sex steroid profiles in maturing female sockeye salmon**

Changes in plasma sex steroids in relation to sexual maturation processes have been reported in sockeye salmon (Schimdt and Idler,1962; Truscott et al., 1986) as well as in other Pacific salmon species (Sower and Schreck, 1982; Young et al., 1983; Fitzpatrick et al., 1986; Dye et al., 1986). Elevated estradiol concentrations are associated with exogenous vitellogenesis (Udea et al., 1984) and reach maximum levels a month prior to ovulation (Ueda et al., 1984; Dye et al., 1986; Fitzpatrick et al., 1986; Truscott et al., 1986; Slater et al., 1994). During the period of vitellogenesis, oocytes develop, gain mass through accumulation of yolk, and progress through several stages based on germinal vesicle (the nucleus of the oocyte) location or diameter of oocyte, including: central germinal vesicle, migrating germinal vesicle, and peripheral germinal vesicle. A precipitous decrease in E2 marks the termination of vitellogenin deposition and the preparation of the oocyte for final maturation and ovulation (Truscott et al., 1986; Kubokawa et al., 1999). As exogenous vitellogenesis concludes, DHP levels in the blood surge, marking the reinitiation of meiosis, characterized by breakdown of the germinal vesicle, the oocyte’s nucleus, followed by ovulation (Sower and Schreck, 1982; Young et al., 1983; Udea et al., 1984; Dye et al., 1986; Fitzpatrick et al., 1986; Truscott et al., 1986; Slater et al., 1994; Kubokawa et al., 1999).

In the context of various maturation stages, concentrations of E2 are high in females with oocytes at with central or migrating germinal vesicles (corresponding to immature and maturing stages), decrease at the peripheral germinal vesicle stage, and remain low in fish that have undergone germinal vesicle breakdown and ovulation (corresponding to mature and spawning stages). Conversely plasma levels of DHP are low at central, migrating, and peripheral germinal vesicle stages and increase dramatically at ovulation (Onuma 2003). These trends are represented graphically based on data from studies correlating sex steroid levels to behavioral stages (immature, maturing, spawning, post-spawning) (Sato, 1997; Leonard, 2002, Fig. C1). Similar patterns and trends were produced from studies examining these sex steroid levels on the basis of geography and migration stage (Onuma, 2009; Ueda, 1998; Fig. C1). In Bristol Bay, fish transit the commercial fishery and enter freshwater with elevated E2 levels during the in-river migration. Fish sampled at spawning streams immediately prior to entry to the spawning grounds are in the final stages of maturation, therefore E2 levels should be declining and 17,20β-DHP levels should be elevated.

Figure S1. Spline curves fit to plasma concentrations of E2 versus 17,20β-DHP as a function of advancing ovarian maturation (top graph) or migration stage (bottom graph ) for coho (*Oncorhynchus kisutch*) and masu samon (*O. masou*). Reproductive maturation stage profiles were reconstructed from multiple year studies (year=2) (Fitzpatrick et al. 1986). Migration stage profiles reconstructed from multiple year study (Onuma et al., 2003), and studies by Onuma et al. (2009) and Ueda et al. (1998).

**References**

Dye, H.M., Sumpter, J.P., Fagerlund, U.H.M., Donalson, E.M., 1986. Changes in reproductive parameters during the spawning migration of pink salmon, Oncorhynchus gorbuscha (Walbaum). J. Fish. Bio. 29, 167-176.

Fitzpatrick M.S., Van Der Kraak, G. Schreck C.B., 1986. Profiles of plasma sex steroids and gonadotropin in Coho salmon *Oncorhynchus kisutch*, during final maturation. Gen. Comp. Endocr. 62, 437-451.

Kubokawa, K., Wantanabe, T., Yoshioka, M., Iwata, M., 1999. Effects of acute stress on plasma cortisol, sex steroid hormone and glucose levels in male and female sockeye salmon during the breeding season. Aquaculture 172, 335-349.

Leonard, J.B.K., Iwata, M., Ueda, H., 2002. Seasonal changes of hormones and sucle enzymes in adult lacustrine masu (Oncorhynchus masou)and sockeye (*Oncorhynchus nerka*) salmon. Fish Physiol. Biochem. 25, 53-163.

Onuma, T., Higashi, Y. Ando, H., Ban. M. Ueda, H., Urano, A. 2003. Year-to-year differences in plasma levels of steroid hormones in pre-spawning chum salmon. Gen. Comp. Endocr. 133, 199-215.

Onuma, T., Sato, S., Katsumata, H., Makino, K., Hu, W., Jodo, A., Davis, A., Dickey, J., Ban, M., Ando, H., Fukuwaka, M., Azumaya, T., Swanson, P., Urano, A., 2009. Activity of the pituitary-gonadal axis is increased prior to the onset of spawning migration of chum salmon. J. Exp. Biol. 212, 56-70.

Sato, A., Ueda, H., Fukaya, M., Kaeriyama, M., Zohar, Y., Urano, A., Yamauchi K., 1997. Sexual differences in homing prfolies and shortening of homin duration in Gonadotropin-relaeading hormone analog implantation in lucustrine sockeye salmon. Zool. Sci. 14, 1009-1014.

Schmidt, P.J., Idler, D.R., 1962. Steriod hormones in the plasma of salmon at various states of maturation. Gen. Comp. Endocr. 2, 204-214.

Slater, C.H., Schreck, C.B., Swanson P., 1994. Plasma profiles of the sex steroids and gonadotropins in maturing female spring Chinook salmon (*Oncorhynchus tshawytscha*). Comp. Biochem. Physiol. 109A(1), 167-175.

Sower, S.A., Schreck, C.B., 1982. Steroid and thyroid hormones during sexual maturation of coho salmon (Oncorhynchus kisutch) in sea water or freshwater. Gen. Comp. Endocr. 47, 42-53.

Truscott, B., Idler, D.R., So, Y.P., Walsh, J.M., 1986. Maturational steroids and gonadotropin in upstream migratory sockeye salmon. Gen. Comp. Endocr. 62, 99-110.

Ueda, H., 1998. Correlations between homing migration and reproduction of chum salmon. North Pacific Anadromous Fish Commission Bulletin No.1, 112-117.

Udea, H., Hiroi, O., Hara, A., Yamauchi, K., Nagahama, Y., 1984. Changes in serum concentrations of steroid hormones, thyroxine, and vitellogenin during spawning migrations of the chum salmon, Oncorhynchus keta. Gen. Comp. Endocr. 53, 203-211.

Young, G., Crim L.W., Kagawa, H., Kambegawa, A., Nagahama, Y., 1983. Plasma 17α,20β-dihydrooxy-4-progen-3-one levels during sexual maturation of amago salmon (Oncorhynchus rhodurus): Correlation with plasma gonadotropin and *in vitro* production by ovarian follicles. Gen. Comp. Endocr. 51, 96-105.
